# Draft genome sequences of two distinct *Clostridioides difficile* isolates coinfecting a patient

**DOI:** 10.1128/mra.01017-24

**Published:** 2024-12-10

**Authors:** Xin Lu, Yu Feng, Xiaohui Wang

**Affiliations:** 1 Center of Infectious Diseases, West China Hospital, Sichuan University, Chengdu, China; 2 Laboratory of Pathogen Research, West China Hospital, Sichuan University, Chengdu, China; 3 Division of Infectious Diseases, State Key Laboratory of Biotherapy, Chengdu, China; University of Maryland School of Medicine, Baltimore, Maryland, USA

**Keywords:** *Clostridioides difficile*, coinfection, whole-genome sequencing

## Abstract

*Clostridioides difficile* is a spore-forming anaerobic Gram-positive bacillus. In this study, two distinct strains of *C. difficile* causing coinfection were isolated from the same fecal sample. Whole-genome sequencing was performed on these two strains to elucidate their genomic characteristics.

## ANNOUNCEMENT

*Clostridioides difficile* is a spore-forming anaerobic Gram-positive bacillus. *C. difficile* infection can be mild to moderate recurrent diarrhea but, in severe cases, may result in ileus and toxic megacolon or even death. Sporadic coinfection cases have been reported with two different strains of *C. difficile* previously (1). However, the genetic relatedness between different *C. difficile* strains during coinfection remains unclear. In this work, we report the draft genomes of two distinct strains isolated from one fecal sample collected from a mild *C. difficile*-infected patient hospitalized for chronic kidney disease and pulmonary infection caused by coronavirus disease 2019 (COVID-19). This study was approved by the Ethics Committee of West China Hospital, with a waiver of informed consent.

After shock with alcohol, fecal specimens were transferred to cycloserine-cefoxitin-fructose agar (CCFA, Oxoid, Basingstoke, UK) plates containing taurocholic acid and anaerobically incubated at 37°C for 48 hours. To purify the colony, isolated colonies were passaged once on duplicated blood agar plates under the same culture conditions. Then, colonies with two different morphological sizes were noticed and identified using MALDI-TOF and PCR targeting the housekeeping gene *tpi* and toxin genes *tcdA, tcdB, cdtA,* and *cdtB* (2, 3). Single colonies were anaerobically incubated overnight at 37°C in brain heart infusion broth, and then DNA was extracted using the QIAamp DNA Mini Kit (Qiagen, Hilden, Germany). The NEBNext Ultra II DNA Library Prep Kit (New England Biolabs, Ipswich, MA, USA) was utilized in conjunction with SPRIselect (Beckman Coulter, Brea, CA, USA) to create libraries targeting 350-bp insert fragments after sonication of the DNA. The prepared libraries were loaded onto the NovaSeq 6000 (Illumina, San Diego, CA, USA) for 150-bp paired-end sequencing. Read pairs were first trimmed to remove adapters, using Trimmomatic v0.39 (4), followed by a coverage reduction to 150× using SeqKit v2.8.2 (5)and genome assembly with SPAdes v3.15.5 (6)in isolate mode. Species identification was confirmed by comparing the average nucleotide identity (ANI) between queries and the type strain of *C. difficile* DSM 1296^T^ (CP084369.1) using FastANI v1.33 (7)with a cutoff of 95%. Multi-locus sequence typing was performed by querying genomes against the corresponding database held on PubMLST (8). Antimicrobial resistance (AMR) genes were identified using AMRFinderPlus v3.11.18 (9). Genome annotation was automatically performed using PGAP v6.5 (10) following submission to NCBI. Default parameters were used for all bioinformatics tools unless otherwise specified.

Strain 2301801 and strain 2301802 were categorized as ST99 and ST11, respectively. Both strains share 3,166 single-nucleotide polymorphisms using Snippy v4.6.0 (https://github.com/tseemann/ snippy). Both strains contain *tcdA* and *tcdB* genes, while strain 2301802 contains additional *cdtA* and *cdtB* genes (Table 1). The genomic assembly data with more than 99.7% genome completeness and AMR genes are shown in Table 1. This data set provides valuable insights for future research into genomic evolution and bacterial interactions, contributing to a deeper understanding of *C. difficile* coinfection.

**TABLE 1:** Key characteristics of two clinical *C. difficile* strains causing coinfection

Strain	2301801	2301802
ST	99	11
Toxin gene type^*a*^	A+B+CDT-	A+B+CDT+
No. of reads (pairs)	4,848,722	4,276,255
No. of bases (bp)	1,454,616,600	1,282,876,500
ANI^*b*^	99.457%	96.118%
Assembly		
No. of contigs	82	77
Largest contig (bp)	706,096	480,668
Total length (bp)	397,4791	4,001,079
GC (%)	28.25	28.75
N50 (bp)	267,208	162,872
No. of coding sequences	3,531	3,572
No. of tRNAs	77	69
No. of rRNAs	23	21
Genes predicted to mediate antimicrobial resistance	*vanR-Cd, vanS-Cd, vanG-Cd, bla_CDD-2_, vanT-Cd, vanZ1*	*erm(B), tet(M), vanZ1, bla_CDD-1_, ant (6)-Ia, sat4, aph(3')-IIIa, tet(O), aad9*
Accession data		
SRA accession no.	SRR29764273	SRR29481513
GenBank accession no.	JBEJVW000000000.1	JBEJVX000000000.1

^*a*^ Toxin profile: presence/absence of toxin gene *tcdA* (A), *tcdB* (B), and *cdtA/B* (binary toxin locus) (CDT).

^*b*^ Compared with the type strain of *C. difficile* DSM 1296^T^ (CP084369.1).

## Data Availability

The SRA and GenBank nucleotide accession numbers of the draft genome sequences of *C. difficile* 2301801 and 2301802 are summarized in Table 1.
